# Electrophilic PPAR***γ*** Ligands Attenuate IL-1***β*** and Silica-Induced Inflammatory Mediator Production in Human Lung Fibroblasts via a PPAR***γ***-Independent Mechanism

**DOI:** 10.1155/2011/318134

**Published:** 2011-06-16

**Authors:** Christopher M. Hogan, Thomas H. Thatcher, Ramil E. Sapinoro, Michael N. Gurell, Heather E. Ferguson, Stephen J. Pollock, Carolyn Jones, Richard P. Phipps, Patricia J. Sime

**Affiliations:** ^1^Division of Pulmonary and Critical Care Medicine, University of Rochester, 601 Elmwood Avenue, P.O. Box 692, Rochester, NY 14642, USA; ^2^Department of Environmental Medicine, University of Rochester, Rochester, NY 14642, USA; ^3^Lung Biology and Disease Program, University of Rochester, Rochester, NY 14642, USA; ^4^Department of Surgery, University of Rochester, Rochester, NY 14642, USA

## Abstract

Acute and chronic lung inflammation is associated with numerous important disease pathologies including asthma, chronic obstructive pulmonary disease and silicosis. Lung fibroblasts are a novel and important target of anti-inflammatory therapy, as they orchestrate, respond to, and amplify inflammatory cascades and are the key cell in the pathogenesis of lung fibrosis. Peroxisome proliferator-activated receptor gamma (PPAR*γ*) ligands are small molecules that induce anti-inflammatory responses in a variety of tissues. Here, we report for the first time that PPAR*γ* ligands have potent anti-inflammatory effects on human lung fibroblasts. 2-cyano-3, 12-dioxoolean-1, 9-dien-28-oic acid (CDDO) and 15-deoxy-Δ^12,14^-prostaglandin J_2_ (15d-PGJ_2_) inhibit production of the inflammatory mediators interleukin-6 (IL-6), monocyte chemoattractant protein-1 (MCP-1), COX-2, and prostaglandin (PG)E_2_ in primary human lung fibroblasts stimulated with either IL-1*β* or silica. The anti-inflammatory properties of these molecules are not blocked by the PPAR*γ* antagonist GW9662 and thus are largely PPAR*γ* independent. However, they are dependent on the presence of an electrophilic carbon. CDDO and 15d-PGJ_2_, but not rosiglitazone, inhibited NF-*κ*B activity. These results demonstrate that CDDO and 15d-PGJ_2_ are potent attenuators of proinflammatory responses in lung fibroblasts and suggest that these molecules should be explored as the basis for novel, targeted anti-inflammatory therapies in the lung and other organs.

## 1. Introduction

Inflammation is associated with many diseases of the lung and can result from immunologic injury, infection, and inhalation of particulate matter. Diseases strongly associated with pulmonary inflammation include asthma, chronic obstructive pulmonary disease (COPD), and silicosis. Inflammation is also associated with an increased susceptibility to developing lung cancers and other malignancies [1–4]. Aside from glucocorticoids, few effective anti-inflammatory agents exist. In this regard, it is important to investigate new anti-inflammatory targets.

Peroxisome proliferator-activated receptor gamma (PPAR*γ*) has emerged as an important potential anti-inflammatory target. PPAR*γ* is a ligand-activated nuclear receptor that binds a variety of endogenous lipids and lipid-derived compounds. Upon ligand binding, PPAR*γ* heterodimerizes with the retinoid X receptor (RXR), translocates to the nucleus, and regulates expression of genes containing PPAR*γ* response elements (PPREs) [5]. PPAR*γ* ligands regulate key cellular processes including differentiation, proliferation, adipogenesis, and insulin sensitization [6, 7]. PPAR*γ* ligands have also been shown to attenuate inflammation in many tissues including skin, liver, kidney, and lung [8–11]. Several types of natural PPAR*γ* ligands exist, including prostaglandins such as 15-deoxy-Δ^12,14^-prostaglandin J_2_ (15d-PGJ_2_), and fatty acids such as lysophosphatidic acid and 15S-hydroxyeicosatetraenoic acid [12–15]. The thiazolidinedione (TZD) class of drugs, including rosiglitazone and pioglitazone, are synthetic PPAR*γ* agonists that are used as insulin sensitizers for type II diabetes [16, 17]. 2-cyano-3, 12-dioxoolean-1, 9-dien-28-oic acid (CDDO) is a novel synthetic triterpenoid PPAR*γ* ligand currently in clinical trials for treatment of several different forms of cancer [18, 19]. 

Rosiglitazone, CDDO and 15d-PGJ_2_ all tightly bind to PPAR*γ* [12, 20, 21], activate PPAR*γ*-dependent transcription [22, 23], and regulate adipogenesis via a PPAR*γ*-dependent mechanism [12, 24, 25]. However, CDDO and 15d-PGJ_2_ also have effects which can be mediated through PPAR*γ*-independent pathways [26–28]. 15d-PGJ_2_ and CDDO contain *α*/*β*-unsaturated ketone rings with electrophilic carbons susceptible to forming covalent bonds with cellular proteins through Michaels addition reactions [18, 29, 30]. Rosiglitazone and other synthetic TZDs lack these electrophilic centers. We have recently demonstrated the importance of these electrophilic carbons in preventing TGF-*β*-induced myofibroblast differentiation [26]. Understanding the molecular pathways modulated by PPAR*γ* ligands will shed light on their potential therapeutic contribution(s) in the control of pulmonary inflammation. 

In addition to their structural role, fibroblasts in the lung act as sentinel cells with significant effector roles in orchestrating and amplifying inflammatory cascades. They become activated when exposed to inflammatory stimuli and produce inflammatory mediators such as IL-6, monocyte chemoattractant protein-1 (MCP-1), cyclooxygenase-2 (COX-2), and PGE_2_ [31–34]. We hypothesized that PPAR*γ* ligands would exhibit anti-inflammatory effects in human lung fibroblasts, and tested this hypothesis using IL-1*β*, a potent proinflammatory cytokines, and silica, an inhaled particulate with strong pro-inflammatory effects on lung fibroblasts [32, 33]. Here, we report for the first time that PPAR*γ* ligands inhibit the inflammatory response of human lung fibroblasts, and do so via a largely PPAR*γ*-independent pathway dependent on a strong electrophilic center.

## 2. Material and Methods

### 2.1. Cells and Cell Culture

Primary human lung fibroblasts were derived from tissue explants obtained from patients undergoing surgical resection for benign hamartoma. This is an abnormal but noncancerous growth within the lung, it is not an inflammatory or fibrotic disease. The tissue pieces used to obtain the fibroblasts were taken from a region of the resected tissue that was most distal to the hamartoma that was anatomically and histologically normal [35]. Patient samples were obtained with approval of the Institutional Review Board of the University of Rochester. These cells are morphologically consistent with fibroblasts [36]. They express collagen and vimentin, and they do not express CD45, factor VIII, or cytokeratin. Fibroblasts were cultured in minimum essential media (MEM, Life Technologies, Gaithersburg, Md, USA) supplemented with 10% fetal bovine serum (FBS, Sigma Aldrich, St. Louis, Mo, USA), 2 mM L-glutamine, penicillin (100 units/mL), streptomycin (100 *μ*g/mL), and amphotericin (0.25 *μ*g/mL) (Life Technologies) at 37°C in 7% CO_2_, as previously described [26]. Cells were used at passages 6–12.

### 2.2. Reagents

PPAR*γ* agonists and related compounds rosiglitazone, 9,10-dihydro-15-deoxy-Δ^12,14^-PGJ_2_ (CAY10410), GW9662, and prostaglandin-A_1_ (PGA_1_) were from Cayman Chemical (Ann Arbor, MI). 2-cyano-3, 12-dioxoolean-1, 9-dien-28-oic acid (CDDO) was obtained from the NIH-RAID Program and Reata Pharmaceuticals (Dallas, Tex, USA) and 15-deoxy-Δ^12,14^-prostaglandin J_2_ (15d-PGJ_2_) was from Biomol (Plymouth Meeting, PA). These compounds were prepared as 10 mM stocks in DMSO and added to cell cultures to the final concentrations indicated. Control wells (media only) were supplemented with DMSO to the same final concentration (0.2%) as treated wells. One hour after pretreatment with PPAR*γ* ligands, inflammatory stimulants interleukin-1*β* (IL-1*β*, R&D Systems, Minneapolis, Minn, USA) and amorphous silica (a generous gift of Dr. David R. Hemenway, University of Vermont) were added to the cell cultures at the final concentrations indicated for 24 hours. Amorphous silica was prepared by baking for 2 hours at 180°C prior to addition to MEM.

### 2.3. Cytotoxicity Assays

Cell viability was assessed by 3-[4,5-dimethylthiazol-2-yl]-2,5-diphenyltetrazolium bromide (MTT) assay [37]. Fibroblasts were plated in triplicate at a density of 5,000 cells per well in 96 well plates and treated with TGF-*β* and PPAR*γ* agonists for 24 h at the indicated concentrations. MTT was added for the final 4 hours. Production of the colored reaction product was measured at 560 nm, and the results were normalized to the negative control wells. LDH activity in culture medium was measured by a commercial assay (Sigma).

### 2.4. Prostaglandin and Cytokine Assays

Primary human lung fibroblasts (100,000 cells/well) were plated in six-well plates (Falcon/Becton Dickson, Franklin Lakes, Nj, USA), serum starved for 48 hours, and treated with IL-1*β* or silica and/or PPAR*γ* agonists as described. PGE_2_ was measured in harvested supernatants using a commercially available competitive enzyme immunoassay (EIA) (Cayman Chemical) [38]. IL-6 and CCL2/MCP-1 in harvested supernatants were determined by ELISA according to the manufacturer's instructions (R&D Systems, Minneapolis, Minn, USA).

### 2.5. Western Blots for Cyclooxygenase-2 (COX-2)

Total cellular protein extracts were prepared from lung fibroblast cultures with 10% Nonidet P-40 (NP-40) lysis buffer supplemented with a protease inhibitor cocktail (Sigma). Lysates were clarified by centrifugation and proteins were quantitated by the bicinchoninic acid (Pierce, Rockford, IL). Typically, 5 *μ*g of total solubilized cellular protein was separated by 10% SDS-PAGE under reducing conditions and transferred to nitrocellulose membranes. Blots were probed with a COX-2 specific monoclonal antibody (Cayman Chemical) using GAPDH (Abcam, Cambridge, Mass, USA) as a loading control. Proteins were visualized with enhanced chemiluminescence (Western Lightning, Perkin-Elmer, Wellesley, MA) [38] and densitometry of the resulting bands was performed using Kodak Molecular Imaging Software (Rochester, NY) normalized to the loading control.

### 2.6. COX-2 Immununfluorescence

Fibroblasts were cultured in eight-well chamber slides (5 × 10^4^ cells/well) in serum-free MEM for 48 hours before treatment. Some cells were pretreated with PPAR*γ* agonists for one hour prior to IL-1*β* (1 ng/mL) treatment for the indicated duration. Cells maintained in serum-free MEM were used as negative controls. Cells were washed in PBS and fixed and permeabilized for 15 minutes at room temperature in BD Cytofix/Cytoperm solution (BD Biosciences, San Diego, Calif, USA). The cells were then washed with BD Perm/Wash buffer (BD Biosciences) in this step and all future washes to maintain permeabilization. Nonspecific sites were blocked with 5% normal goat serum (Life Technologies) in BD Perm/Wash buffer for 15 minutes at room temperature or 4°C overnight. Monoclonal mouse anti-human COX-2 antibody was diluted in 1% normal goat serum in BD Perm/Wash buffer and incubated with the cells overnight at 4°C. Cells were washed and Alexa Fluor 488 (green-) tagged goat anti-mouse IgG (Invitrogen) diluted in 1% normal goat serum in BD Perm/Wash buffer was added to the cells for one hour at room temperature. Cells were washed a coverslipped with ProLong Antifade with DAPI (Invitrogen).

### 2.7. NF-*κ*B Luciferase Assay

We generated human lung fibroblast strains that stably expressed a mammalian codon-optimized firefly luciferase reporter gene under the transcriptional control of NF-*κ*B response elements using NF-*κ*B Cignal Lenti Reporter lentiviral particles (SABiosciences Corporation, Frederick, Md, USA) at an MOI = 25. Two days post-infection, growth medium was removed and replaced with growth medium containing 1 *μ*g/mL puromycin. Puromycin-resistant clones were identified and expanded for propagation. To investigate NF-*κ*B activity, primary human lung fibroblasts expressing NF-*κ*B-Luc (3,000 cells/well) were plated in 96-well plates, serum starved for 48 hours and treated with IL-1*β* and/or PPAR*γ* agonists as described. Cells were lysed in 1x Passive Lysis Buffer and mixed with Luciferase Assay Reagent II as instructed by the manufacturer (Promega, Madison, Wis, USA). Equal protein quantities were used in luciferase assays; results were reported in relative light units.

### 2.8. Statistics

All data are expressed as mean ± SD. A one-way analysis of variance (ANOVA) with Tukey post-test were used to establish statistical significance. Results were considered significant if *P* < .05.

## 3. Results

### 3.1. PPAR*γ* Ligands Inhibit IL-1*β*-Induced Inflammatory Cytokine Production in Human Lung Fibroblasts

**Figure 1:**
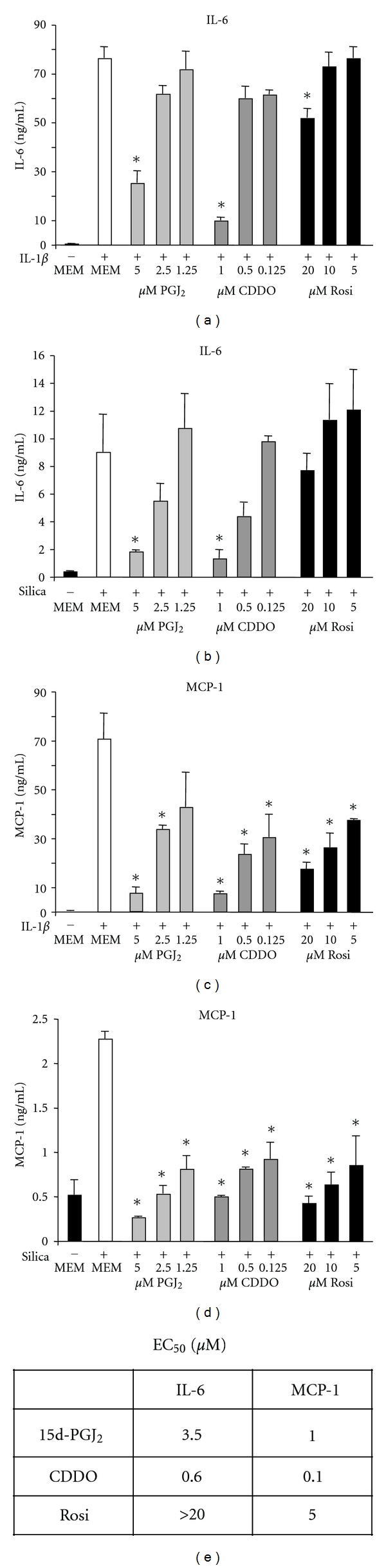
PPAR*γ* ligands attenuate IL-6 and MCP-1 production by human lung fibroblasts induced by IL-1*β* or crystalline silica. Primary human lung fibroblasts were pretreated with the indicated concentrations of rosiglitazone (Rosi), CDDO or 15d-PGJ_2_ (PGJ_2_) for 1 hour and then cotreated with 1 ng/mL IL-1*β* (a, b) or 10 *μ*g/mL crystalline silica (c, d) for 24 hours. Supernatants were harvested and cytokine concentrations were determined by ELISA. Results are mean ± standard error for quadruplicate wells and are representative of 2 independent experiments that yielded similar results. (e) The EC_50_ was determined from the given data and averaged for each cytokine. Data were analyzed by one-way ANOVA. * = *P* < .05 compared to stimulus alone (MEM).

To determine the efficacy of selected PPAR*γ* ligands in inhibiting production of inflammatory mediators in lung fibroblasts, primary human lung fibroblasts were pretreated with rosiglitazone, CDDO, or 15d-PGJ_2_ for 1 hour and then co-treated with a powerful pro-inflammatory stimulus, IL-1*β* (1 ng/mL), for 24 hours. IL-1*β* strongly induced the pro-inflammatory mediators IL-6 and MCP-1 (Figure 1). Rosiglitazone, CDDO, and 15d-PGJ_2_ all show dose-dependent inhibition of cytokine production and significantly inhibited the release of these mediators. We also investigated an alternative inflammatory stimulus, crystalline silica, which we have previously reported is a potent pro-inflammatory stimulus in human lung fibroblasts [32]. Silica also strongly induced the production of IL-6 and MCP-1, which was inhibited by the PPAR*γ* ligands in a dose-dependent manner (Figures 1(c) and 1(d)). Interestingly, all 3 ligands were 4-5-fold more effective at blocking MCP-1 than IL-6 (Figure 1(e)).

**Figure 2:**
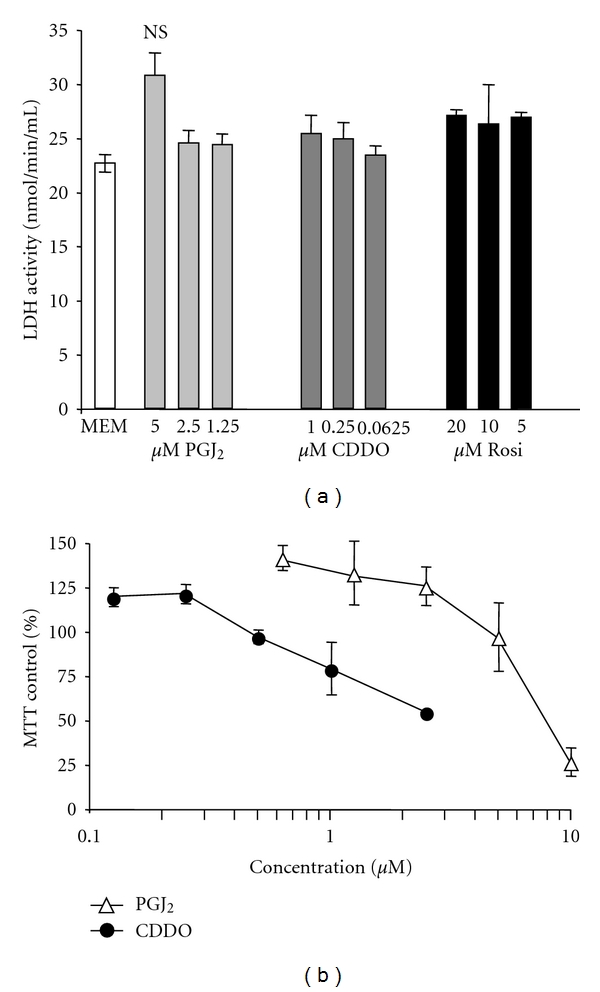
The PPAR*γ* ligands are not overtly cytotoxic at the doses used. (a) Primary human lung fibroblasts were treated for 24 hours with the indicated concentrations of CDDO, or 15d-PGJ_2_ (PGJ_2_), and LDH released into the media was measured by commercial LDH activity assay. There were no significant differences between any of the treatment groups compared to MEM control. Results are mean ± standard deviation for triplicate wells and are representative of 2 independent experiments that yielded similar results. (b) Primary human lung fibroblasts were plated in a 96-well plate and treated with the indicated concentrations of CDDO or 15d-PGJ_2_ (PGJ_2_). Cell viability was determined after 24 hours by MTT assay. The results shown are the mean ± standard deviation of quadruplicate wells and are normalized to untreated control wells.

It should be noted that the maximum dose used for each compound is the highest dose that does not cause overt cyotoxicity (Figure 2 and data not shown). Rosiglitazone is at least 10-fold less effective than CDDO or 15d-PGJ_2_ and is a very poor inhibitor of IL-6 release even at the maximum tolerable dose of 20 *μ*M.

### 3.2. PPAR*γ* Ligands Inhibit IL-1*β*-Induced Upregulation of COX-2 and PGE_2_

**Figure 3:**
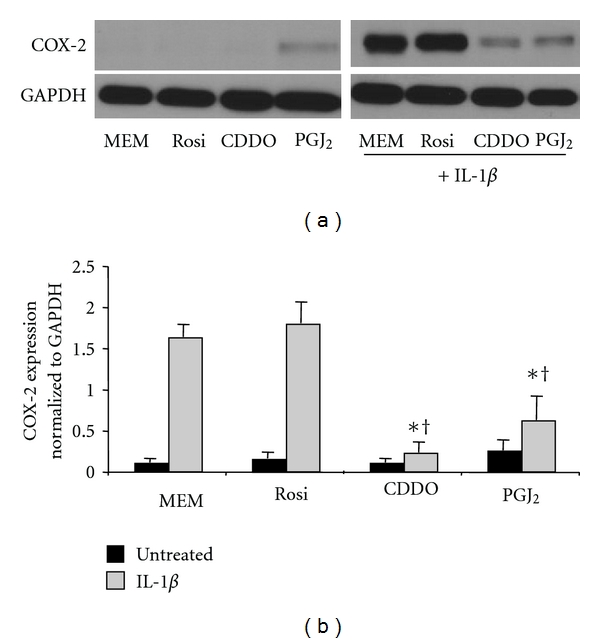
CDDO and 15d-PGJ_2_ attenuate IL-1*β*-induced COX-2 production in human lung fibroblasts. Primary human lung fibroblasts were pretreated with 20 *μ*M rosiglitazone (Rosi), 1 *μ*M CDDO, or 5 *μ*M 15d-PGJ_2_ (PGJ_2_) for 1 hour and then cotreated with 1 ng/mL of IL-1*β* for 24 hours. Protein lysates were harvested and Western blot analysis was performed by probing for protein expression of COX-2 and GAPDH (for normalization). (a) Representative samples are shown. (b) Quadruplicate samples were analyzed by densitometry and normalized to GAPDH. COX-2 expression was significantly reduced in CDDO and 15d-PGJ_2_ treated cells compared to IL-1*β* alone (*P < .001). CDDO and 15d-PGJ_2_ were significantly more potent than rosiglitazone (^†^ *P* < .001). Results are mean ± standard deviation for quadruplicate wells and are representative of 3 independent experiments that yielded similar results. Data were analyzed by ANOVA.

To further evaluate the potential antiinflammatory actions of PPAR*γ* ligands, lung fibroblasts were treated with IL-1*β* and PPAR*γ* ligands, and expression of COX-2 was determined by Western blot. As expected, IL-1*β* strongly induces COX-2 (Figure 3). CDDO and 15d-PGJ_2_ significantly inhibited IL-1*β*-induced COX-2 production. However, rosiglitazone failed to suppress COX-2 induction in human lung fibroblasts. 

**Figure 4:**
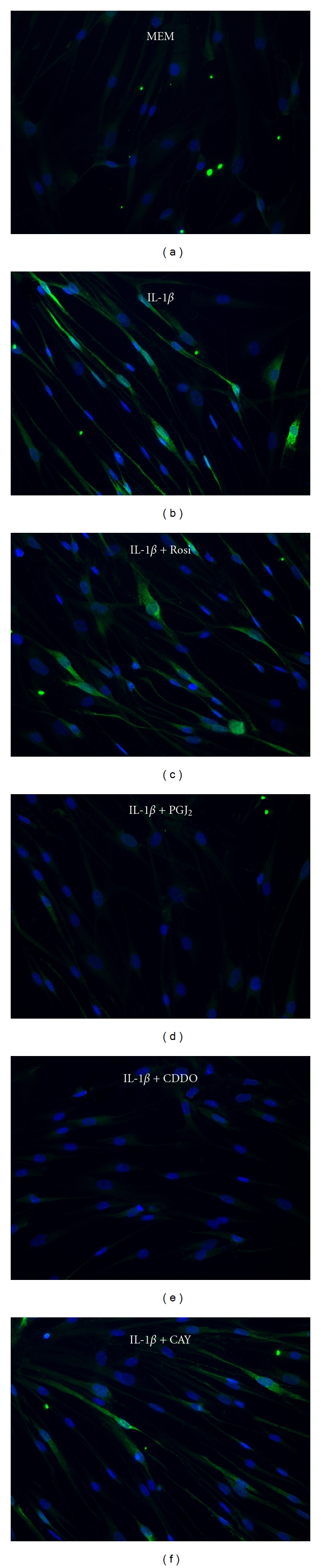
Immunofluorescence of human lung fibroblasts demonstrates CDDO and 15d-PGJ_2_ attenuate IL-1*β*-induced COX-2 expression. Fibroblasts were pretreated with PPAR*γ* agonists for 1 hour prior to IL-1*β* (1 ng/mL) treatment for 24 hours. Cells were fixed and permeabilized and probed for COX-2 protein. COX-2 was visualized with Alexa Fluor 488 (green) and cell nuclei with DAPI (blue). (a) MEM control; (b) IL-1*β*; (c) IL-1*β* + 20 *μ*M rosiglitazone; (d) IL-1*β* + 1 *μ*M CDDO; (e) IL-1*β* + 5 *μ*M 15d-PGJ_2_, (f) IL-1*β* + 5 *μ*M CAY10410. IL-1*β*-induced COX-2 was inhibited by CDDO (d) and 15d-PGJ_2_ (e).

We also performed immunofluorescence to confirm the induction of COX-2 protein and to localize the expression of COX-2 in IL-1*β*-treated lung fibroblasts. COX-2-specific staining demonstrated that untreated fibroblasts expressed minimal COX-2 at baseline (Figure 4(a)). Upon treatment with IL-1*β*, cytoplasmic COX-2 production was markedly upregulated (Figure 4(b)). Rosiglitazone failed to suppress IL-1*β*-induced COX-2 upregulation (Figure 4(c)). In contrast, however, both CDDO and 15d-PGJ_2_ suppressed cytoplasmic expression of COX-2 in IL-1*β*-treated fibroblasts (Figures 4(d) and 4(e)). CAY10410, a structural analogue of 15d-PGJ_2_, did not suppress IL-1*β*-induced COX-2 upregulation (Figure 4(f)), suggesting structural differences between the ligands account for their anti-inflammatory actions.

**Figure 5:**
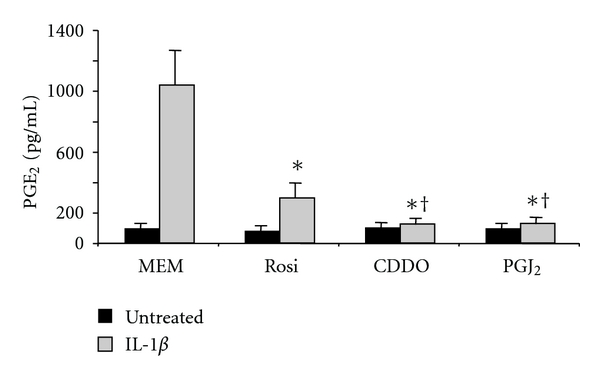
PPAR*γ* ligands attenuate IL-1*β*-induced PGE_2_ production in human lung fibroblasts. Primary human lung fibroblasts were pretreated with 20 *μ*M rosiglitazone (Rosi), 1 *μ*M CDDO, or 5 *μ*M 15d-PGJ_2_ (PGJ_2_) and then cotreated with IL-1*β* for 24 hours as previously described. Supernatants were harvested, and PGE_2_ was determined by EIA. PGE_2_ production is significantly reduced in PPAR*γ* ligand-treated fibroblasts compared to IL-1*β* alone (*P < .001). CDDO and 15d-PGJ_2_ were significantly more potent than rosiglitazone (^†^ *P* < .05). Results are mean ± standard deviation for quadruplicate wells and are representative of 3 independent experiments that yielded similar results. Data were analyzed by ANOVA.

COX-2 mediates the first step in the conversion of arachidonic acid to prostaglandins. The immunomodulatory prostaglandin PGE_2_, a product of this reaction, was measured in lung fibroblast culture supernatants following treatment with PPAR*γ* ligands and IL-1*β*. Consistent with the COX-2 results, CDDO and 15d-PGJ_2_ inhibited IL-1*β*-induced production of PGE_2_ by greater than 90% compared to controls treated with IL-1*β* alone (Figure 5). Rosiglitazone also inhibited IL-1*β*-induced production of PGE_2_, but was less effective than CDDO and 15d-PGJ_2_.

### 3.3. Suppression of Inflammatory Mediators by PPAR*γ* Ligands in Human Lung Fibroblasts Occurs via a PPAR*γ*-Independent Mechanism

**Figure 6:**
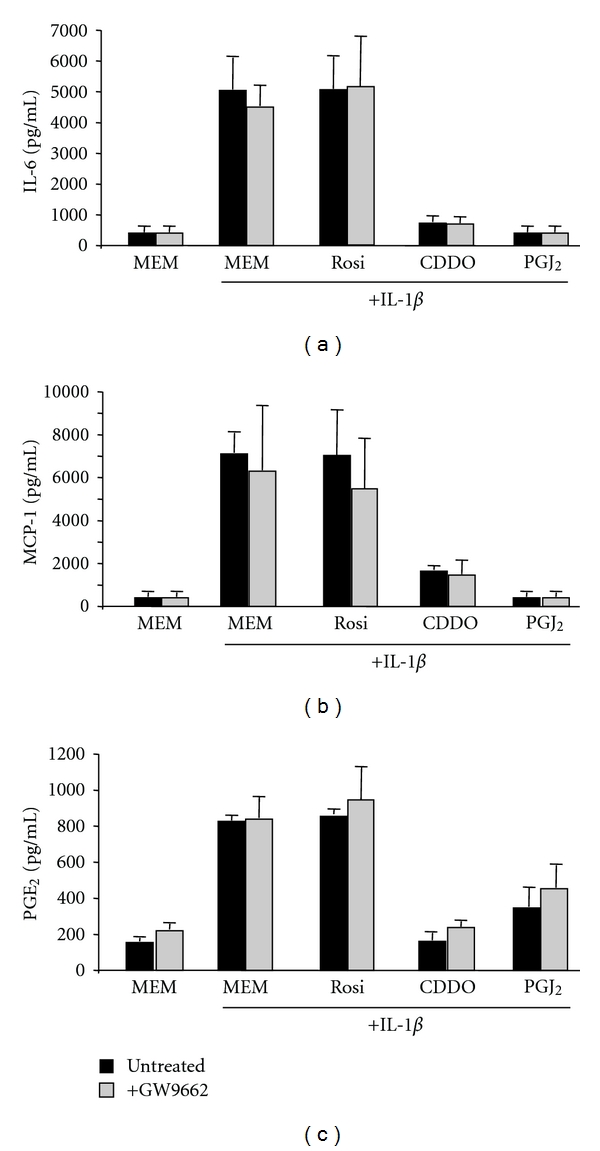
The PPAR*γ* antagonist GW9662 does not inhibit the anti-inflammatory effects of CDDO and 15d-PGJ_2_. Primary human lung fibroblasts were pretreated with 1 *μ*M GW9662 for 3 hours, then PPAR*γ* ligands were added for 1 hour, then 1 ng/mL IL-1*β* was added for 24 hours. Supernatants were harvested and proinflammatory mediators were measured by ELISA or EIA. (a) IL-6, (b) MCP-1, (c) PGE_2_. Pretreatment with GW9662 did not significantly alter the attenuation of pro-inflammatory mediator production by CDDO or 15d-PGJ_2_ alone. Results are mean ± standard deviation for quadruplicate wells and are representative of 2 independent experiments that yielded similar results.

We used a pharmacological approach to determine whether the anti-inflammatory actions of PPAR*γ* ligands are dependent on or independent of PPAR*γ*. GW9662 is an irreversible PPAR*γ* antagonist that covalently binds to a cysteine residue in the ligand binding site of PPAR*γ* [39]. GW9662 inhibits PPAR*γ* agonist-driven adipogenesis, which is a completely PPAR*γ*-dependent process [24]. Primary human lung fibroblasts were pretreated for 4 hours with GW9662 and one hour with CDDO or 15d-PGJ_2_, followed by IL-1*β*. IL-1*β* strongly induced IL-6, MCP-1 and PGE_2_ compared to MEM control (Figures 6(a)–6(c)). As previously shown, CDDO and 15d-PGJ_2_ significantly inhibited the IL-1*β*-induced production of these inflammatory mediators. GW9662 did not reverse the suppressive effects of CDDO and 15d-PGJ_2_ ligands on cytokine and PGE_2_ production (Figures 6(a)–6(c)). This indicates that PPAR*γ* is not essential for the anti-inflammatory effects of these ligands, and that PPAR*γ* independent pathways are therefore likely important.

### 3.4. A Strong Electrophilic Center Is Important for PPAR*γ* Ligand-Mediated Suppression of Inflammation in Human Lung Fibroblasts

**Figure 7:**
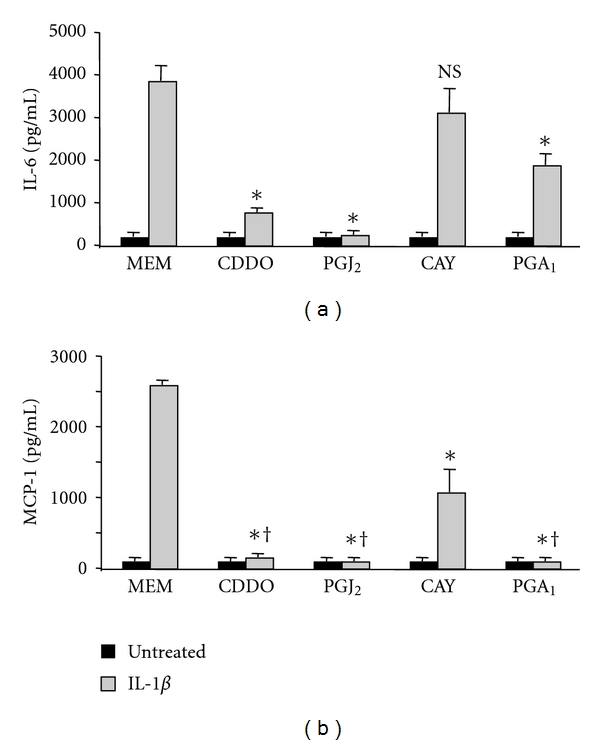
A strong electrophilic carbon is necessary for PPAR*γ* ligand-mediated attenuation of inflammation in IL-1*β*-treated human lung fibroblasts. Primary human lung fibroblasts were pretreated with 1 *μ*M CDDO, 5 *μ*M 15d-PGJ_2_ (PGJ_2_), 5 *μ*M CAY10410, (CAY) or 15 *μ*M PGA_1_ for 1 hour and then co-treated with 1 ng/mL of IL-1*β* for 24 hours. Supernatants were harvested, and cytokine concentrations were measured by ELISA. (a) CDDO, 15d-PGJ_2_, and PGA_1_, but not CAY10410, significantly inhibited production of IL-6 (*P < .01). (b) CDDO, 15d-PGJ_2_, PGA_1_, and CAY10410 all significantly reduced MCP-1 production (*P < .01). CDDO, 15d-PGJ_2_, and PGA_1_ were significantly more potent than CAY10410 (^†^ *P* < .01). Results are mean ± standard deviation for quadruplicate wells and are representative of 3 independent experiments.

CDDO and 15d-PGJ_2_ contain *α*/*β*-unsaturated ketone rings with electrophilic carbons that can form covalent bonds with free sulfhydryls in cellular proteins [40, 41]. CAY10410 (9,10-dihydro-15-deoxy-Δ^12,14^-PGJ_2_) is a structural analog of 15d-PGJ_2_ that lacks the unsaturated ketone containing the electrophilic carbon. To investigate the importance of the electrophilic center in suppressing inflammatory endpoints, we compared the ability of 15d-PGJ_2_ and CAY10410 to inhibit the pro-inflammatory effects of IL-1*β* on human lung fibroblasts. CAY10410 treatment resulted in a small reduction in IL-1*β*-induced IL-6 production that was not statistically significant, and a 60% reduction in MCP-1 production compared to 98% inhibition by 15d-PGJ_2_ (Figure 7). 

To further investigate the importance of the electrophilic center, we tested another prostaglandin that is also a potent electrophile, PGA_1_. PGA_1_ was partially effective at inhibiting IL-1*β*-induced production of IL-6 and completely effective in blocking MCP-1 production (Figure 7).

### 3.5. A Strong Electrophilic Center Is Important for PPAR*γ* Ligand-Mediated Suppression of NF-*κ*B in Human Lung Fibroblasts

**Figure 8:**
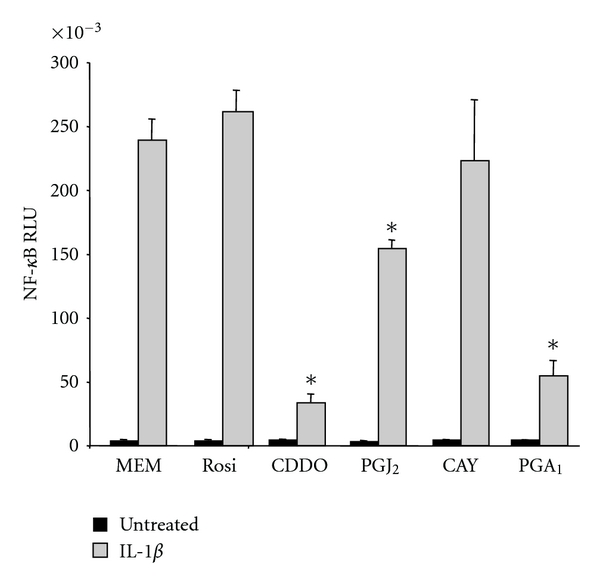
CDDO and PGA_1_ inhibit IL-1*β*-induced NF-*κ*B transcriptional activity in human lung fibroblasts. Primary human lung fibroblasts were transfected with a luciferase reporter, then pretreated with 20 *μ*M rosiglitazone (Rosi), 1 *μ*M CDDO, 5 *μ*M 15d-PGJ_2_ (PGJ_2_), 5 *μ*M CAY10410 (CAY), or 15 *μ*M PGA_1_ and cotreated with of IL-1*β* for 24 hours as described. NF-*κ*B-dependent luciferase activity was measured in lysates as described, normalized to protein concentration, and expressed as relative light units (RLU). NF-*κ*B activation is significantly reduced in PPAR*γ* ligand-treated fibroblasts compared to IL-1*β* alone (*P < .01). Results are mean ± standard deviation for quadruplicate wells and are representative of 2 independent experiments that yielded similar results.

To better understand the mechanism involved in PPAR*γ* ligand-mediated immune suppression, we investigated the effect of PPAR*γ* ligands on the activation of NF-*κ*B, a transcription factor that regulates the expression of numerous pro-inflammatory mediators. Primary human lung fibroblasts were transfected with an NF-*κ*B luciferase reporter construct, and treated with PPAR*γ* ligands and IL-1*β*. CDDO, 15d-PGJ_2_, and PGA_1_, but not CAY10410 or rosiglitazone, significantly decreased IL-1*β*-induced NF-*κ*B luciferase activity (Figure 8).

## 4. Discussion

Inflammation is associated with many diseases of the lung including asthma, chronic obstructive pulmonary disease (COPD), silicosis, and lung cancer [1–4]. Aside from glucocorticoids, there are few effective anti-inflammatory therapies; therefore, the development of novel therapies that have the potential to alleviate pulmonary diseases associated with inflammatory etiologies is a priority.

PPAR*γ* ligands are receiving increasing attention as potential anti-inflammatory therapeutics because of their anti-inflammatory properties in a variety of tissues in vivo and cells in vitro [42]. The anti-inflammatory effects of PPAR*γ* ligands have not previously been reported in human lung fibroblasts, a sentinel cell of inflammatory cascades in the lung [31, 34, 43, 44]. Here, we report that PPAR*γ* ligands have potent anti-inflammatory effects in human lung fibroblasts exposed to divergent inflammatory stimuli, and that the mechanism is largely PPAR*γ*-independent.

To induce a pro-inflammatory response in human lung fibroblasts, we used two different inflammatory stimuli. IL-1*β* is an acute phase inflammatory cytokine, while silica is a particulate that has potent proinflammatory effects when inhaled and is capable of causing both acute and chronic inflammatory lung disease [32, 33]. Both IL-1*β* and silica induced the inflammatory mediators IL-6 and MCP-1, which were inhibited by CDDO, rosiglitazone, and 15d-PGJ_2_ (Figure 1). Interestingly, rosiglitazone was much less effective at inhibiting IL-6 and MCP-1, with an EC_50_ 5–10-fold higher than 15d-PGJ_2_ and at least 30-fold higher than CDDO. CDDO and 15d-PGJ_2_, but not rosiglitazone, also blocked upregulation of COX-2 and PGE_2_ (Figures 3 and 4). This is in agreement with our previous finding that rosiglitazone is less effective than CDDO or 15d-PGJ_2_ at inhibiting the pro-fibrotic effects of TGF-*β* in lung fibroblasts [26], and suggests that there are significant differences in the mechanism of action between rosiglitazone and CDDO and 15d-PGJ_2_.

Rosiglitazone, CDDO and 15d-PGJ_2_ all tightly bind the PPAR*γ* receptor [12, 20, 21], activate PPAR*γ*-dependent transcription [22, 23], and promote adipogenesis via a solely PPAR*γ*-dependent mechanism [12, 25]. However, in addition to stimulating PPAR*γ*-dependent transcriptional changes, CDDO and 15d-PGJ_2_ are reported to have effects that are mediated through PPAR*γ*-independent pathways [26, 28, 45]. To determine whether CDDO, and 15d-PGJ_2_ might be acting via a PPAR*γ*-independent mechanism, we used a pharmacological approach to block PPAR*γ*. GW9662 is an irreversible competitive PPAR*γ* antagonist that covalently binds to a cysteine residue in the ligand binding domain of PPAR*γ* [46]. GW9662 is a highly effective inhibitor of PPAR*γ*-dependent processes including differentiation of osteoclasts and activation of hepatic stellate cells [47, 48]. We have previously reported that rosiglitazone, CDDO and 15d-PGJ_2_ drive the differentiation of fibroblasts to adipocytes. GW9662 at 1 *μ*M completely inhibits this effect, demonstrating that this compound is effective at blocking the PPAR*γ*-dependent actions of these PPAR*γ* ligands [24]. Here, GW9662 did not reverse the anti-inflammatory effects of CDDO and 15d-PGJ_2_ (Figure 6), indicating that the anti-inflammatory effects of CDDO and 15d-PGJ_2_ on human lung fibroblasts are largely independent of the PPAR*γ*-dependent transcriptional pathway. Rosiglitazone was such a poor inhibitor of the inflammatory effects of IL-1*β* that it was not possible to show a reversal of inhibition by GW9662, which would be expected if rosiglitazone acted by a purely PPAR*γ*-dependent mechanism.

Comparing the chemical structures of rosiglitazone, CDDO, and 15d-PGJ_2_, it is notable that CDDO and 15d-PGJ_2_ have strong electrophilic carbons, whereas rosiglitazone does not. 15d-PGJ_2_ has one *α*/*β*-unsaturated ketone ring with an electrophilic carbon capable of forming covalent bonds through Michael addition reactions [49], whereas CDDO has two [18, 30]. We have recently demonstrated the importance of these electrophilic carbons in preventing TGF-*β*-inducedmyofibroblast differentiation [26, 50]. We hypothesize that the electrophilic carbons of CDDO and 15d-PGJ_2_ are also important for their anti-inflammatory effects. To test this hypothesis, we used CAY10410, a structural analog of 15d-PGJ_2_ that lacks the *α*/*β*-unsaturated ketone, and PGA_1_, another electrophilic prostaglandin. In lung fibroblasts stimulated with IL-1*β*, CAY10410 did not inhibit COX-2 upregulation or IL-6 production and was half as effective as 15d-PGJ_2_ at blocking MCP-1 production (Figures 4 and 7). On the other hand, PGA_1_ significantly attenuated IL-6 and completely blocked production of MCP-1 (Figure 7). Because CAY10410 has an identical structure to 15d-PGJ_2_ except for the electrophilic carbon, the fact that CAY10410 lacks the effects of 15d-PGJ_2_ strongly suggests that the electrophilic centers present in CDDO and 15d-PGJ_2_ are critical for mediating their maximal anti-inflammatory therapeutic potential. CDDO and 15d-PGJ_2_, but not rosiglitazone or CAY10410, significantly inhibited IL-1*β*-induced NF-*κ*B activity (Figure 8). 

The molecular targets of CDDO and 15d-PGJ_2_ in inflammation are not completely known. 15d-PGJ_2_ can bind to the NF-*κ*B components I*κ*B and p65 [50]. Another candidate is the transcription factor Nrf2, which regulates anti-oxidant and anti-inflammatory pathways. CDDO and 15d-PGJ_2_ activate Nrf2 in mouse cells and human cancer cells [51, 52]. However, these compounds do not activate Nrf2 in human lung fibroblasts [27, 53]. We have previously reported that CDDO activates AP-1 transcriptional activity in human lung fibroblasts [27]. However, AP-1 is a promoter, rather than an inhibitor of inflammation, and AP-1 activation leads to upregulation of IL-6 via NF-*κ*B [54]. We hypothesize that these electrophilic compounds suppress inflammation and activate AP-1 via different pathways, and that the anti-inflammatory effects are stronger and override the potentially proinflammatory effects of AP-1 activation. 

In addition to PPAR*γ*-independent effects, PPAR*γ* ligands have anti-inflammatory effects that are moderated via a PPAR*γ*-dependent mechanism. This PPAR*γ*-dependent mechanism can be accessed by TZDs such as rosiglitazone and pioglitazone [9, 55–57], and indeed, rosiglitazone has limited anti-inflammatory properties in this report. However, while TZDs are currently used clinically as insulin sensitizers in type 2 diabetes, they have a complex sideeffect profile including edema, weight gain, bone weakness, and potentially an increased risk of cardiovascular disease [58–60], that may limit their widespread use as anti-inflammatory therapies. Although TZDs have high binding affinity for PPAR*γ* they lack electrophilic centers and are thus unable to access PPAR*γ*-independent anti-inflammatory pathways that use this mechanism [27, 49, 61, 62]. We suggest that additional research on the PPAR*γ*-independent anti-inflammatory activities of CDDO and 15d-PGJ_2_, including identification of additional targets beyond NF-*κ*B, should lead to development of novel compounds with greater specificity for the anti-inflammatory targets of PPAR*γ* ligands but decreased binding of PPAR*γ* itself, with fewer resulting side-effects. As CDDO is orally active, has a long half-life, and is currently in clinical trials as an anticancer therapy, it may be a useful platform for derivatization and further study. Further development of small compounds with strong electrophilic centers is warranted as these drugs may be effective anti-inflammatory treatments for human lung diseases.
